# Antibodies Targeting KSHV gH/gL Reveal Distinct Neutralization Mechanisms

**DOI:** 10.3390/v14030541

**Published:** 2022-03-05

**Authors:** Thomas Fricke, Anna K. Großkopf, Armin Ensser, Marija Backovic, Alexander S. Hahn

**Affiliations:** 1 Junior Research Group Herpesviruses, German Primate Center—Leibniz-Institute for Primate Research, 37077 Göttingen, Germany; tfricke@dpz.eu (T.F.); agrosskopf@dpz.eu (A.K.G.); 2 Institute for Clinical and Molecular Virology, Friedrich-Alexander-Universität Erlangen-Nürnberg, 91054 Erlangen, Germany; armin.ensser@fau.de; 3 Structural Virology Unit, Institut Pasteur, CNRS UMR3569, 75724 Paris, France; marija.backovic@pasteur.fr

**Keywords:** KSHV, HHV-8, neutralizing antibodies, gH/gL, herpesvirus entry, fusion

## Abstract

Kaposi’s sarcoma herpesvirus (KSHV) is associated with a significant disease burden, in particular in Sub-Sahara Africa. A KSHV vaccine would be highly desirable, but the mechanisms underlying neutralizing antibody responses against KSHV remain largely unexplored. The complex made of glycoproteins H and L (gH/gL) activates gB for the fusion of viral and cellular membranes in all herpesviruses. KSHV gH/gL also interacts with cellular Eph family receptors. To identify optimal antigens for vaccination and to elucidate neutralization mechanisms, we primed mice with recombinantly expressed, soluble gH/gL (gHecto/gL) that was either wildtype (WT), lacking defined glycosylation sites or bearing modified glycosylation, followed by boosts with WT gHecto/gL. We also immunized with a gL-gHecto fusion protein or a gHecto-ferritin/gL nanoparticle. Immune sera neutralized KSHV and inhibited EphA2 receptor binding. None of the regimens was superior to immunization with WT gHecto/gL with regard to neutralizing activity and EphA2 blocking activity, the gL-gHecto fusion protein was equally effective, and the ferritin construct was inferior. gH/gL-targeting sera inhibited gB-mediated membrane fusion and inhibited infection also independently from receptor binding and gL, as demonstrated by neutralization of a novel KSHV mutant that does not or only marginally incorporate gL into the gH/gL complex and infects through an Eph-independent route.

## 1. Introduction

Kaposi’s sarcoma herpesvirus (KSHV) is the causative agent of Kaposi’s sarcoma (KS) [1,2]. KSHV is also associated with B cell malignancies such as primary effusion lymphoma [3] and a variant of multicentric Castleman’s disease [4], and Kaposi sarcoma inflammatory cytokine syndrome [5,6]. Recently, KSHV was also found to be associated with osteosarcoma [7]. The KSHV-associated disease burden is, without doubt, the largest in Sub-Saharan Africa, where seroprevalence exceeds 80% in some regions [2,8,9]. The situation in Africa is compounded by HIV, even though KS was observed before the HIV epidemic, and also as a pediatric tumor preferentially affecting boys [10,11]. In a relatively recent study from Malawi, 9% of KS cases occurred in HIV-negative individuals [12]. Other factors that contribute to KSHV-associated pathogenesis may be malaria [13] or genetic polymorphism [14,15].

KSHV, like all herpesviruses, possesses a conserved set of three glycoproteins (GP), glycoprotein (g) B (gB), gH, and gL, that together form the so-called core fusion machinery (CFM) [16], which is critical for infection by herpesviruses. gB is the fusion executor of the herpesviral entry machinery [16], and the activity of gB is proposed to be controlled by the gH/gL complex [16]. But in KSHV, gH/gL has functions beyond its role in membrane fusion and interacts with a number of cellular proteins such as heparan sulfate proteoglycans [17], EphA2 receptor [18]—an important determinant of KSHV infection and likely pathogenesis [14]—and with several other Eph receptors [19,20,21].

KSHV further has a unique glycoprotein, K8.1, which is positionally conserved with Epstein–Barr virus (EBV) gp350. The K8.1 GP is the immunodominant KSHV surface antigen with regard to antibody responses [22], which is why it is widely used as an antigen for KSHV serology. Antibodies to K8.1 were shown to neutralize infection of tonsillar B cells and of a B cell line [23]. In a recent report, the gH/gL complex was identified as the major target of neutralizing antibodies in sera of KSHV-infected individuals [24]. We sought to determine the optimal immunization regimen and the most potent antigen construct to induce neutralizing antibodies by immunizing mice with a panel of recombinant soluble gHecto/gL variants. We evaluated the antibody response in relation to the ability of recovered sera to neutralize KSHV infection and to block the interaction with EphA2 to elucidate the mechanism of neutralization. We separately analyzed the effect of sera on membrane fusion as a proxy for the antibodies blocking the CFM, which consists of gH/gL and gB. 

## 2. Materials and Methods

### 2.1. Cells 

Human embryonic kidney (HEK) 293T cells (RRID:CVCL_0063) (laboratory of Tobias Moser) and SLK cells (RRID:CVCL_9569) (NIH AIDS Research and Reference Reagent program) were cultured in Dulbecco’s modified Eagle medium (DMEM), high glucose, GlutaMAX, 25 mM HEPES (Thermo Fisher Scientific, Dreieich, Germany) supplemented with 10% fetal calf serum (FCS) (Thermo Fisher Scientific) and 50 μg/mL gentamycin (PAN Biotech, Aidenbach, Germany). The GnTI-HEK 293S cells [25] (a kind gift from Stefan Pöhlmann) were additionally supplemented with 1 mM sodium pyruvate (Thermo Fisher Scientific).

### 2.2. Plasmids 

The pcDNA3.1-KSHV-gL was ordered from GeneScript and was codon-optimized based on (ref|NC_009333|). The pcDNA3.1_KSHVgL_N118Q/N141Q was cloned based on pcDNA3.1-KSHV-gL by using “Round the Horn” site-directed mutagenesis. PCR product clean-up was performed using the NucleoSpin Gel and PCR Clean-up Kit (Macherey-Nagel, Düren, Germany), followed by phosphorylation using T4 PNK (NEB) in T4 Ligation buffer (NEB) and ligation using QuickLigase (NEB) according to manufacturer’s instruction.

The pcDNA6-KSHV-gHecto-TEV-TandemStrep_N46Q/N54Q was cloned based on pcDNA6-KSHV-gHecto-TEV-TandemStrep by using “Round the Horn” site-directed mutagenesis. pcDNA6-KSHV-gHecto-TEV-TandemStrep encodes the first 704 amino acids of codon-optimized KSHV gH (ref|AF210726.1|) (purchased from GeneArt) fused to a sequence coding for a tobacco etch virus mosaic protease (TEV) recognition site and a tandem strep tag (ENLYFQSTSAWSHPQFEKGGGSGGGSGGGSAWSHPQFEK). The pcDNA6-KSHV gHecto-ferritin was cloned by fusing the Helicobacter pylori-bullfrog hybrid ferritin [26] with a stop codon into pcDNA6-KSHV-gHecto-TEV-TandemStrep to the C-terminus of the KSHV gH ectodomain (aa 1-704) by Gibson assembly. The Helicobacter pylori-bullfrog hybrid ferritin consists of (residues 2–9 of bullfrog (*Rana catesbeiana*) ferritin (UniProt: P07797 with a N8Q mutation to abolish a potential N-glycosylation site) and *H. pylori* nonheme ferritin (UniProt: Q9ZLI1, residues 3–167 with an I7E mutation) and was synthesized by Twist Bioscience. 

The pcDNA6-KSHV_gL-gHecto-TEV-TandemStrep expression plasmid was cloned by inserting KSHV gL without its signal peptide (aa 21-167) in frame into pcDNA6-KSHV-gHecto-TEV-TandemStrep after the signal peptide (aa 1-22). Sequencing of the plasmid showed a single nucleotide insertion in the linker sequence between the two strep tags leading to a frameshift and an early termination (ENLYFQSTSAWSHPQFEKGGGSGGGSGGGVSLVTSTV). As the mutation is behind the TEV cleavage site and the protein was efficiently purified by Strep-Tactin™XT Superflow (IBA), we decided to continue with this plasmid. The KSHV gH-ASAELAAN construct was generated based on pcDNA6aV5-KSHV-gH I49AE52AF53A using “Round the Horn” site-directed mutagenesis. 

The pcDNA6a EphA2ectodomain_1-436_His was cloned from pcDNA6a EphA2ectodomain_1-534_His by using “Round the Horn” site-directed deletion. pcDNA6a EphA2ectodomain_1-534_His encodes the first 534 amino acids of EphA2 (ref|NM_004431|) fused to six histidines in a pcDNA6 backbone. The pet11_HIS_STREP_EndoH was ordered from GeneScript and was codon-optimized based on Endo-beta-N-acetylglucosaminidase (aa 32-313) (ref|WP_087792540|) with an N-terminal 6xHis and Strep-tag. The expression construct for KSHV gHecto-FcStrep (original name of the construct in prior publications was gH-FcStrep) as well as pcDNA6-KSHV-gL-Flag, pcDNA6aV5-KSHV-gH, pcDNA6aV5-KSHV-gH I49AE52AF53A [27], Gal4-TurboGFP-Luc, and the plasmids for the fusion assay were described previously [28,29] (See Table 1 for oligonucleotide sequences).

### 2.3. Recombinant Proteins 

Recombinant EndoH and TEV were expressed in *E. coli* BL21 Star overnight in TB medium and induced with 1 mM IPTG. The cells were opened by sonication and centrifuged at 50,000× *g*. The supernatant with the TEV protease was passed over 1 mL of a Ni-NTA Agarose (Macherey-Nagel) matrix in a gravity flow Omniprep column (BioRad, Feldkirchen, Germany). Bound protein was washed with approximately 50 mL TBS (150 mM NaCl, 50 mM Tris-HCl, pH 7.6) and eluted in 1 mL fractions with 500 mM Imidazole in TBS. The supernatant with the EndoH was passed over 2 mL of a Strep-Tactin™XT Superflow (IBA) in a gravity flow Omniprep column (BioRad). Bound protein was washed with approximately 50 mL TBS (150 mM NaCl, 50 mM Tris-HCl, pH 7.6) and eluted in 1 mL fractions with 80 mM Biotin in TBS. Protein-containing fractions were pooled and concentrated via VivaSpin columns with 30 kDA molecular weight cutoff (MWCO) (Sartorius, Göttingen, Germany). Protein concentration was determined by absorbance at 280 nm. Aliquots were shock frozen at 2.5 mg/mL and stored at −80 °C.

Recombinant KSHV gHecto-Strep/gL protein complex, gHecto(N46Q/N54D)-Strep/gL(N118Q/N141Q) protein complex, gHecto-ferritin/gL protein complex, gL-gHecto-Strep fusion protein, and gHecto-FcStrep/gL were purified under native conditions from HEK 293T or GnTI-HEK 293S cell culture supernatant. HEK 293T or GnTI-HEK 293S cells were transfected using PEI transfection [30]. The protein-containing cell culture supernatant was filtered through 0.22 μm PES membranes (Millipore), concentrated using VIVAFLOW 50R (Sartorius). The supernatants of KSHV gHecto-Strep/gL protein complex, gHecto(N46Q/N54D)-Strep/gL(N118Q/N141Q) protein complex, and gL-gHecto-Strep fusion protein were passed over 2 mL of Strep-Tactin™XT Superflow (IBA) in a gravity flow Omniprep column (BioRad). Bound protein was washed with approximately 50 mL TBS (150 mM NaCl, 50 mM Tris-HCl, pH 7.6) and eluted in 1 mL fractions with 80 mM Biotin in TBS. The supernatant of gHecto-ferritin/gL protein complex were passed over 1 mL of a Galanthus nivalis agglutinin-immobilized agarose resin (EY Laboratories) in a gravity flow Omniprep column (BioRad). Bound protein was washed with approximately 5 mL TBS (150 mM NaCl, 50 mM Tris-HCl, pH 7.6) and eluted in 1 mL fractions with 200 mM α-Mannose in TBS. Protein-containing fractions were pooled and concentrated via VivaSpin columns with 30 kDa MWCO (Sartorius). The proteins, except for gHecto-ferritin, were then digested overnight with 50 µg TEV protease and in the case of the protein complex for Group 4, also with 50 µg EndoH and separated on a HiPrep 16/60 Sephacryl S300HR column (GE) using an Äkta Avant (GE) chromatography system. An SEC standard curve using bovine thyroglobulin (670 kDa), bovine gamma globulin (150 kDa), chicken albumin (44.3 kDa), and bovine pancreas ribonuclease A (13.7 kDa) (Sigma Cat. Nr. 69385) was generated (MW = 4 × 10^7^ × e^−0.086×Retention_Volume[mL]^) to calculate the approximate molecular size of the complexes. For all recombinant proteins, protein-containing fractions were pooled and buffer exchange to PBS via 30 kDa MWCO VivaSpin columns (Sartorius) was performed. Protein concentration was determined by absorbance at 280 nm. Aliquots were shock frozen and stored at −80 °C.

Recombinant EphA2 ectodomain protein (aa 1-436) was purified under native conditions by Ni-NTA chromatography from 293T cell culture supernatant. 293T cells were transfected with pcDNA6-ectoEphA2-6XHis using PEI transfection [30]. The protein-containing cell culture supernatant was filtered through 0.22 μm PES membranes (Millipore, Darmstadt, Germany), concentrated using VIVAFLOW 50R (Sartorius) and passed over 1 mL of a Ni-NTA Agarose (Macherey-Nagel) matrix in a gravity flow Omniprep column (BioRad). Bound protein was washed with approximately 50 mL TBS (150 mM NaCl, 50 mM Tris-HCl, pH 7.6) and eluted in 1 mL fractions with 500 mM imidazole in TBS. Protein-containing fractions were pooled, concentrated via VivaSpin columns (Sartorius) (30 kDa MWCO), and separated on a HiPrep 16/60 Sephacryl S300HR column (GE) using an Äkta Avant system (GE). Protein-containing fractions were pooled and then buffer exchange to PBS via 30 kDa MWCO VivaSpin columns (Sartorius) was performed. Protein concentration was determined by absorbance at 280 nm. Aliquots were frozen and stored at −80 °C. 

### 2.4. Immunoprecipitation, SDS Polyacrylamide Electrophoresis and Western Blot

SDS polyacrylamide electrophoresis (PAGE) was performed using 8–16% gradient gels (Thermo Fisher Scientific, Dreieich, Germany). Colloidal Coomassie staining was performed using Imperial Protein Stain (Thermo Fisher Scientific, Dreieich, Germany). For pulldown, 293T cells were transfected with pcDNA3, pcDNA6aV5-KSHV-gH, or pcDNA6aV5-KSHV-gH-ASAELAAN and pcDNA6-KSHV-gL-Flag using PEI in a 1:10 ratio (gH/gL). Lysates of 293T cells transfected with the respective expression constructs for gH-V5/gL-Flag complexes and lysates from non-transfected cells were prepared in NP40 lysis buffer (1% Nonidet P40 Substitute (Sigma-Aldrich), 150 mM NaCl (Sigma-Aldrich, St. Louis, MO, USA), 50 mM HEPES pH 7.5 (VWR), 1 mM EDTA (Amresco, Solon, OH, USA) with freshly added Protease Inhibitor Cocktail (Amresco). Subsequently, lysates were incubated with 1 μg V5-tag antibody (BioRad, Feldkirchen, Germany) and ProteinG sepharose (GenScript, Piscataway, NJ, USA) overnight at 4 °C with agitation. ProteinG beads were collected by brief centrifugation and washed three times in NP40 lysis buffer. Precipitates were heated in 2× SDS sample buffer (95 °C, 5 min). Western blotting was performed as described previously [27] using the respective antibodies (Table 2).

### 2.5. Production of KSHV and KSHV gH-ASAELAAN 

Eph-interaction-negative KSHV (KSHV gH-ASAELAAN) was generated using a two-step, markerless λ-red-mediated BAC recombination strategy as described by [31] and harbors amino acid substitutions L47A, I49A, E52A and F53A within the ORF22 open reading frame endoding gH in KSHV BAC16 [32]. In short, recombination cassettes were generated from the pEPKanS template by polymerase chain reaction (PCR) with S7 Fusion High-Fidelity DNA Polymerase (Biozym, Hessisch Oldendorf, Germany) using long oligonucleotides (Ultramers; purchased from Integrated DNA Technologies (IDT)) GCTCCGCCACGCAGCTCATCAATGGGAGAACCAACGCATCCGCTGAACTGGCAGCAAACGGCACTAGTTTTTTTCTAGGATGACGACGATAAGTAGGG and GCTCCGCCACGCAGCTCATCAATGGGAGAACCAACGCATCCGCTGAACTGGCAGCAAACGGCACTAGTTTTTTTCTAGGATGACGACGATAAGTAGGG. Recombination cassettes were transformed into BAC16-carrying *Escherichia coli* strain GS1783, followed by kanamycin selection, and subsequent second recombination under 1% L(+)arabinose (Sigma-Aldrich)-induced I-SceI expression. Colonies were verified by PCR of the mutated region followed by sequence analysis (Macrogen Europe B.V., Amsterdam, Netherlands), pulsed-field gel electrophoresis and restriction fragment length polymorphism. For this purpose, bacmid DNA was isolated by standard alkaline lysis from 5 mL liquid cultures. Subsequently, the integrity of bacmid DNA was analyzed by digestion with restriction enzyme XhoI and separation in 0.8% PFGE agarose (Bio-Rad) gels and 0.5× TBE buffer by pulsed-field gel electrophoresis at 6 V/cm, 120-degree field angle, switch time linearly ramped from 1 s to 5 s over 16 h (CHEF DR III, Bio-Rad). Infectious KSHV reporter viruses were produced as described previously [27]. The sequence of the virus was verified by Illumina sequencing of DNA prepared by Proteinase K digest and phenol/chloroform extraction and ethanol precipitation from a viral stock as described previously [33].

### 2.6. Immunization

Immunization of mice was performed by Davids Biotechnologie GmbH (Röntgenstraße 3, Regensburg, Germany). Five female mice (BALB/c) with an age of 7 weeks were immunized with each antigen complex. The mice were housed by Davids in the animal laboratory under the following environmental conditions: (temperature: 21 ± 1 °C, relative humidity: 55 ± 10% °C, ventilation: 21 ± 3-air change per hour filtered on G4 filters). Daylight and artificial lighting with a circadian cycle of 12 h of light (7 a.m.–7 p.m.). Before the start of the experiments, mice were acclimatized to these conditions for a period of 2 weeks. They were fed ad libitum by a standard pellet diet. Water was distributed ad libitum. Before the immunization start, serum samples (preimmune serum) were prepared from each mouse. For the immunizations of one mouse 20 μg of the protein complexes with a concentration of 0.5 mg/mL was mixed with an equal volume of 10 mg/mL aluminium hydroxide adjuvant (Alhydrogel adjuvant 2%, InvivoGen, Toulouse, France). The immunization was performed immediately after the production of the antigen-adjuvant-mixture. A total of 5 immunizations for each mouse was performed with an antigen amount of 10 μg (40 μL) for each inoculation. The antigen emulsion was applied intramuscular at two different sites. Blood samples (test bleed) were taken at day 35. The final bleed was performed at day 63.

### 2.7. Enzyme-Linked Immunosorbent Assay (ELISA) 

Antibody binding to recombinant gHecto/gL was determined by ELISA by Davids Biotechnologie GmbH. NUNC MicroWell 96-well Polystyrol Plates were coated with 500 ng recombinant gHecto/gL at 10 μg/mL in 0.1 M Bicarbonate pH 9 for 24 h at 4 °C. The wells were blocked with 200 μL Blocking Solution (Davids Biotechnologie Cat. No. D302) for 1 h at room temperature. After four washes with 250 μL Wash Buffer (Cat. No. D303) at room temperature, the wells were incubated with mouse sera for 24 h at 4 °C. The plates were washed four times with 250 μL Wash Buffer (Cat. No. D303) at room temperature. Bound protein was detected via Anti-Mouse-Antibody conjugated to alkaline phosphatase (1:10,000, 6 h, room temperature). The ELISA plate was washed four times with 250 μL Wash Buffer (Cat. No. D303) followed by a wash with 100 mM diethanolamine pH 9.5, 0.5 mM MgCI_2_, 0.1 mM ZnCl_2_. Pierce 1 Step pNPP Solution (Pierce Cat. No. 37621) was added and the plates were imaged on ELISA Reader (BioTek 800). 

Inhibition of the interaction of gH/gL with the EphA2 receptor by mouse sera was measured by EphA2 binding ELISA. F96 Maxisorp Nunc-Immuno Plates (Thermo Fisher Scientific) were coated with recombinant EphA2 ectodomain protein at 10 μg/mL in PBS overnight at 4 °C. After three washes with PBS-T, the wells were blocked with 10% FBS in PBS for 2 h, and afterwards, incubated with the heat-inactivated mouse sera in 10% FBS in PBS and addition of KSHV gHecto-FcStrep/gL to 1 μg/mL in 10% FBS in PBS. The plates were incubated for 2 h at room temperature and washed three times with PBS-T. Bound protein was detected via the C-terminal Strep Tag using horseradish peroxidase (HRP)-coupled StrepTactin secondary reagent (IBA). After three washes, 3,3′,5,5′-Tetramethylbenzidin (TMB) substrate (Thermo Fisher Scientific) was added and the reaction was stopped by adding 100 mL 1 M HCl. The plates were imaged on a Biotek Synergy 2 plate reader. Three independent experiments were performed. Each experiment was normalized to bound gHecto-FcStrep/gL to EphA2 without the heat-inactivated mouse sera and afterwards averaged for each mouse serum.

### 2.8. Fusion Assay 

On day 1, 293T target cells were transfected overnight with a plasmid encoding a Gal4 response element driven TurboGFP-luciferase reporter (Gal4-TurboGFP-Luciferase). The 293T effector cells were transfected either with empty vector or with expression plasmids for gH_KSHV_, gL_KSHV_, gB_RRV_, and VP16-Gal4 transactivator plasmid using PEI in a 1:10:2:2 ratio. On day 2, 16 h after transfection, the medium on the cells was completely removed and exchanged with fresh medium. 24 h after transfection, the effector cells were trypsinized and seeded in 96-well plates at 50,000 cells/well. On day 3, the medium on the 293T effector cells was removed and exchanged to 50 μL fresh DMEM supplemented with 1% FCS and 50 μg/mL gentamycin, with or without heat-inactivated mouse sera, and incubated for 30 min. The target cells were trypsinized and added to the effector cells. After 48 h, cells were lysed in 65 μL 1× Luciferase Cell culture lysis buffer (E1531, Promega) for 20 min at room temperature and centrifuged for 10 min at 4 °C. 50 μL of each cell lysate was used to measure luciferase activity using the Beetle-Juice Luciferase Assay (PJK Biotech) according to manufacturer’s instructions on a Biotek Synergy 2 plate reader. Three independent experiments were performed. Each experiment was normalized to fusion signal of 293T effector cells transfected just with empty vector and VP16-Gal4 fused with 293T target cells and afterwards averaged for each mouse sera. 

### 2.9. EphA2 Blocking Experiment

For blocking assays, SLK cells were plated at 50,000 cells/cm^2^ and infected one day after plating. For the block with soluble ephrins, cells were pre-incubated with ephrin4-Fc fusion protein (R&D Systems, Minneapolis, MN, USA) at 1.25-fold the final concentration of 2 μg/mL for 30 min at room temperature followed by addition of KSHV in 1/5th of the final volume. Block of KSHV infection with soluble EphA2-Fc decoy receptor was assayed by infection with virus inocula that were pre-incubated with the soluble EphA2-Fc at 40 nM at room temperature for 30 min. PBS and Fc protein were used as controls. EphA2-Fc decoy receptor and Fc protein was produced as described previously [27]. 24 h post-infection cells were harvested by brief trypsinization, followed by the addition of 5% FCS in PBS to inhibit trypsin activity, spun down (1200 rpm, 10 min), washed once with PBS, re-pelleted, and fixed in PBS supplemented with 2% formaldehyde (Carl Roth, Karlsruhe, Germany). A minimum of 10,000 cells was analyzed per sample for GFP expression on an LSRII flow cytometer (BD Biosciences, Heidelberg, Germany). Data was analyzed using Flowing Software (Version 2.5).

### 2.10. Infection Assays and Flow Cytometry 

For infection assays, cells were plated at 50,000 cells/cm^2^ (SLK, 293T). At 6 h after plating, the KSHV virus was incubated for 30 min with or without heat-inactivated mouse sera before being added to the cells. The cells were harvested 48 h post-infection by brief trypsinization, followed by the addition of one volume 5% FCS in PBS to inhibit trypsin activity and fixed in PBS supplemented with 4% formaldehyde (Carl Roth). 10,000 cells were analyzed per sample for GFP expression on an ID7000™ Spectral Cell Analyzer flow cytometer (Sony Biotechnology, San Jose, CA, USA). Data was analyzed using ID7000™ Spectral Cell Analyzer (Sony Biotechnology). Three independent experiments were performed for KSHV and KSHV gH-ASAELAAN. Each experiment was normalized to SLK cells without immune sera infected with the respective virus, and afterwards, averaged for each mouse sera.

### 2.11. Statistical Analysis

Statistical analysis and data visualization was performed with GraphPad PRISM version 9.3.1.

## 3. Results

### 3.1. Immunization with Different Recombinant gH/gL Protein Complexes Elicits Binding Antibody Responses

**Figure 1:**
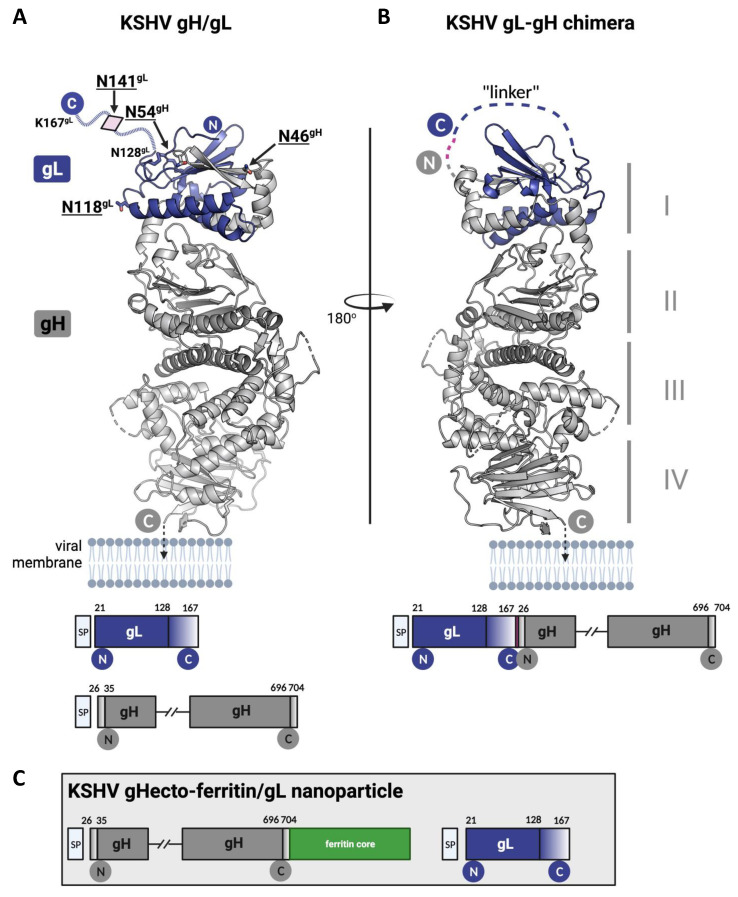
Design of KSHV gHecto/gL immunogens. The gH/gL complex (**A**) and the gL-gH chimera (**B**) are shown on the left and right panels, respectively, with the gHecto-ferritin/gL construct (**C**) in the inlet; I-IV indicate domains; gH is colored in grey and gL in purple, with their N- and C-termini represented as circles containing the letters N and C, respectively. The models are based on the experimentally determined structure of KSHV gH ectodomain/gL complex (PDB:7B7N). The termini of gL (res 129–167) and gH (res 26–34 and 697–704) are not resolved in the structure indicating flexible regions. In the left panel, the putative location of the gL C-terminus (dotted purple line) is shown, with the glycosylation site N141 represented as a pink diamond. The remaining N-glycosylation sites—residues N118 in gL and N46 and N54 in gH—have their side chains represented as stick models. The linker (dashed line on the right panel) that connects the gL and gH in the chimera is formed by the flexible C-terminus of gL (res 129–167) that is joined to flexible N-terminus of gH (res 26–35) by a TGA sequence (highlighted in magenta). SP stands for signal peptide, which was a part of the expression construct.

**Figure 2:**
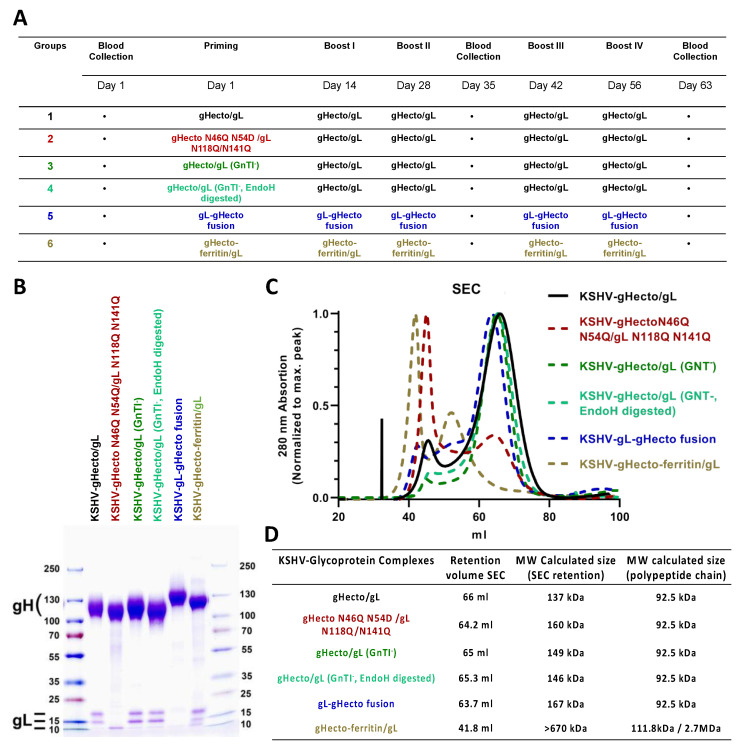
Immunization schedule and gH/gL protein complexes used for immunization. (**A**) Immunization schedule. (**B**) The indicated KSHV gHecto/gL protein complexes were expressed in HEK 293T or GnTI-HEK 293S cells, purified, resolved by SDS PAGE, and stained with colloidal Coomassie. Purified complexes presented a KSHV gH band between 110 and 140 kDa and the three different glycosylation forms of gL between 10 and 20 kDa. (**C**) SEC Chromatograms (HiPrep 16/60 Sephacryl S300HR column (GE)) of the indicated KSHV gHecto/gL protein complexes. (**D**) The retention volume of the different KSHV gHecto/gL complexes and the calculated molecular weight.

Mice were immunized with the recombinant gHecto/gL complexes indicated in Figure 1, using a heterologous or homologous prime-boost regimen (Figure 2A). We specifically did not immunize for the whole series with the glycosylation mutants, but used them as primes. Many viruses evolve glycosylation patterns that can shield antigenically vulnerable sites from the immune response, and, e.g., a recent study by Zhou et al. [34] suggested that a strategy of priming with glycosylation-deficient HIV-1 env may elicit antibodies to otherwise shielded epitopes. The hypothesis behind the heterologous prime boost was that removing some of the glycosylation—in particular that on domain I of gH and in gL (Figure 1A)—would allow for better priming of antibodies targeting these regions that are possibly otherwise partially shielded by glycans, and to then boost B cells that were primed to recognize the partially shielded sites also in the complex bearing WT glycosylation. To this end, we either introduced mutations in domain I of gH and gL (Group 2, Figure 2A), produced the proteins in cells that are deficient for complex glycosylation (GnTI-HEK 293S, Group 3, Figure 2A), or treated such protein with the glycosydase EndoH to remove the glycosylations enzymatically (Group 4, Figure 2A), which should theoretically result in only very little remaining glycosylation, although this may depend on the accessibility of the individual glycosylation sites for the EndoH enzyme in the native protein and result only in a modest shift in the molecular weight of gH (Figure 2B). Prime with these proteins was always followed by boosts with WT, i.e., fully glycosylated, gHecto/gL. For comparison, we immunized with a complete series of primes and boosts with WT gHecto/gL (Group 1, Figure 2A). We also immunized (prime and boosts) with a gL-gHecto fusion protein (Figure 1B; Group 5, Figure 2A) and a gHecto-ferritin fusion protein co-expressed with gL (gHecto-ferritin/gL, Figure 1C; Group 6, Figure 2A), as similar EBV ferritin-based constructs, which assemble into large spheres consisting of 24 subunits, were reported to elicit potent humoral response [35,36]. The gL-gH fusion strategy is not expected to disturb gH/gL folding as the N-terminus of gH and the C-terminus of gL, through which the two proteins are joined (Figure 1B), are flexible and exposed according to the X-ray structure of the KSHV gH/gL complex [37]. The approximate molecular weight of the protein complexes was verified by SDS-PAGE analysis (Figure 2B) and confirmed by size exclusion chromatography (SEC) (Figure 2C,D), comparing SEC elution of the protein complexes to that of a set of protein standards (Figure 2D). SEC indicated apparent molecular weights (MW) between 137 and 167 kDa for the different gHecto/gL complexes and above 670 kDa (the MW of the largest standard for this type of column) for the oligomeric gHecto-ferritin/gL complex, in keeping with expectations. While SEC allows only rough estimation of the MW of the gHecto/gL complexes with different glycosylation content, it demonstrated clearly that the gHecto/gL and gL-gHecto fusion protein complexes are monomeric in solution, while the gHecto-ferritin/gL construct formed large assemblies, consistent with ferritin-mediated self-assembly into nanoparticles. 

**Figure 3:**
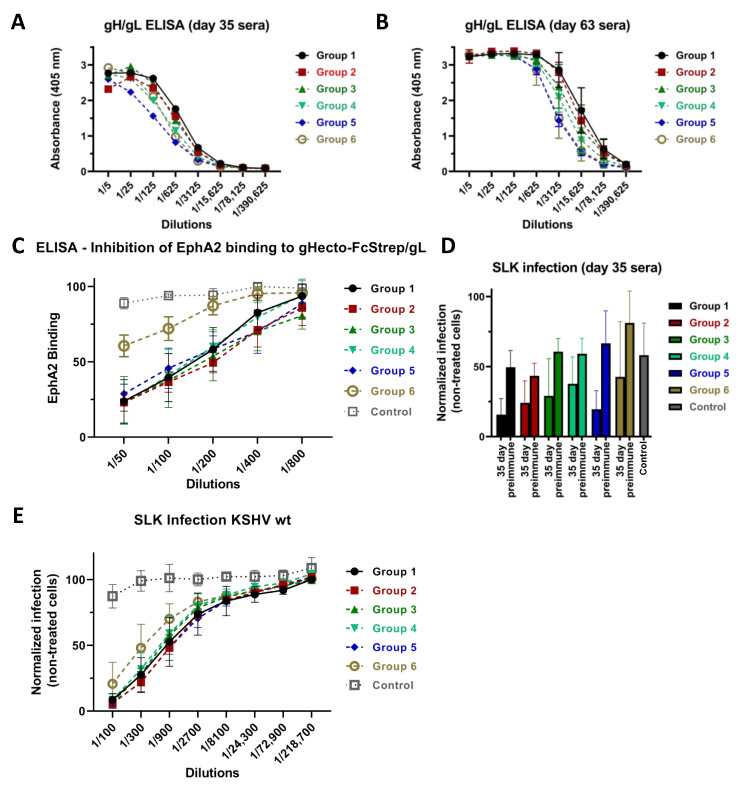
gH/gL binding, inhibition of EphA2 binding, and neutralization. (**A**) gHecto/gL-binding activity of pooled sera from each group at 35 days post start of the immunization schedule measured by ELISA. (**B**) gHecto/gL-binding activity averaged per group from individual sera at 63 days post start of the immunization schedule. Error bars indicate the standard deviation in each group. Group 3 (*p* < 0.005), 4 (*p* < 0.0001), 5 (*p* < 0.0001) and 6 (*p* < 0.0001) sera were significantly less reactive than Group 1 sera at 63 days (two-way ANOVA, Dunnet’s method for correction of multiple comparisons). (**C**) Sera raised against the different KSHV gHecto/gL complexes block gH/gL binding to immobilized EphA2 as measured by ELISA. Sera from Group 6 inhibited EphA2 binding significantly less potently than sera from Groups 1, 2, 3, 4, and 5 (*p* < 0.0001; two-way ANOVA; Tukey’s method for correction for multiple comparisons). All sera inhibited binding significantly as compared to control (*p* < 0.01, two-way ANOVA; Tukey’s method for correction for multiple comparisons). The experiment was performed three times, values were normalized to binding without mouse serum, and normalized values were averaged for each serum. Values of the five sera of each group were then averaged and are shown. Error bars indicate the standard deviation in each group. (**D**) KSHV neutralizing activity of mice sera at 35 days. No significant difference between the groups was observed at 1/100 dilution by one-way ANOVA at day 35 (infection was normalized to infection with preimmune mouse sera; sera from individual mice were treated as biological replicates and averaged values of the five sera of each group are shown; error bars represent the standard deviation). (**E**) KSHV neutralizing activity of mice sera at 63 days. The Sera from Group 1 inhibited significantly more potently than Group 6 sera at 63 days (*p* < 0.0001 extra sum-of-squares F test after non-linear fit for different IC50, inhibitor vs normalized response model; no correction for multiple comparisons of Group 1 to the other groups). The experiment was performed three times; infection was normalized to infection without mouse serum and averaged for each serum. Sera from individual mice were treated as biological replicates and averaged values of the five sera of each group are shown. Error bars represent the standard deviation. Sera from all groups inhibited significantly compared to the control sera (*p* < 0.0001; two-way ANOVA; Dunnett’s method for correction for multiple comparisons).

We first analyzed the antibody responses elicited by the different immunization regimens with regard to binding of recombinant gHecto/gL complex immobilized on ELISA plates. Compared to Group 1, the regimen with fully glycosylated WT gHecto/gL complex, sera from all other groups exhibited a trend towards lower reactivity after the second boost (Figure 3A). This pattern remained stable also at the end of the immunization series (Figure 3B), when all but Group 2 sera were significantly less reactive than Group 1 sera, even if the differences were small. In general, immunization with WT gHecto/gL complex elicited slightly higher ELISA activities than immunization with all other constructs. Of note, the ELISA antigen was the fully glycosylated WT complex, so the highest reactivity against this antigen was probably to be expected for immunization with the exact same antigen.

### 3.2. Immunization with Recombinant gH/gL Elicits Antibodies That Block EphA2 Binding

We next analyzed whether antibodies elicited by immunization with the different gHecto/gL complexes inhibit the interaction of gH/gL with the EphA2 receptor. We measured binding of soluble WT gHecto-FcStrep/gL protein to recombinant soluble EphA2 ectodomain, that had been immobilized on ELISA plates in the presence of anti-gH/gL sera from the different immunization regimens (Figure 3C). With the exception of the sera from the gHecto-ferritin/gL group, the other sera efficiently blocked EphA2 binding to a similar degree.

### 3.3. Neutralizing Activity Is Induced by All Tested gH/gL Preparations

To compare neutralizing activity elicited by the different immunization regimens, SLK cells were infected with KSHV, derived from BAC16 [32] and expressing a GFP reporter gene, after preincubation of the virus with the heat-inactivated sera. We did not observe any major differences between the groups at 35 days (Figure 3D) and only minor differences at 63 days (Figure 3E). Sera from all groups exhibited similar neutralizing activity on SLK cells, with the exception of Group 6 sera (gHecto-ferritin/gL), which were significantly less potent.

### 3.4. Antibodies That Do Not Block gH/gL Interactions with Eph Family Receptors or Target gL Contribute to Virus Neutralization

**Figure 4:**
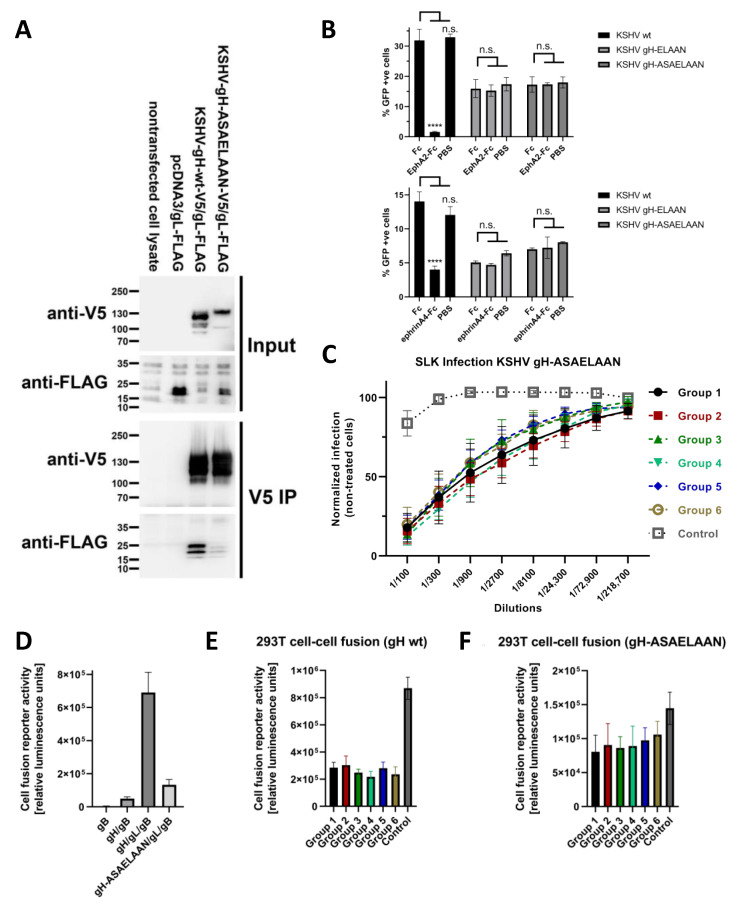
Inhibition of an Eph-detargeted KSHV mutant by sera directed against gH/gL. (**A**) V5 immunoprecipitation of KSHV gH-V5/gL-FLAG and KSHV gH-ASAELAAN-V5/gL-FLAG. The samples were analyzed by Western blot and detected with the indicated antibodies. (**B**) KSHV gH-ASAELAAN is refractory to inhibition with soluble EphA2-Fc decoy receptor (upper panel) or inhibition with soluble ephrinA4-Fc (lower panel). The experiment was performed in triplicate (**** indicates *p* < 0.0001 compared to Fc; two-way ANOVA, Sidak correction for all multiple comparisons). (**C**) Neutralizing activity of mice sera at 63 days against KSHV gH-ASAELAAN. No significant differences were observed between Groups 2–6 and Group 1 (extra sum-of-squares F test after non-linear fit for different IC50, inhibitor vs normalized response model; no correction for multiple comparisons). The experiment was performed three times; infection was normalized to infection without mouse serum and averaged for each serum. Sera from individual mice were treated as biological replicates and averaged values of the five sera of each group are shown. Error bars represent the standard deviation. Sera from all groups inhibited significantly compared to the control sera (*p* < 0.0001; two-way ANOVA; Dunnett’s method for correction for multiple comparisons). (**D**) Cell–cell fusion assay. Cells transfected with KSHV gH-ASAELAAN/gL/RRV gB fuse 5 fold less with the target cells than KSHV gH/gL/RRV gB. Error bars represent the standard deviation. (**E**) Cell–cell fusion assay with KSHV gH/gL/RRV gB expressing effector cells. All groups inhibited fusion significantly (*p* < 0.0001; one-way ANOVA) compared to the control sera. Results were averaged for sera from each group. Error bars represent the standard deviation. (**F**) Cell–cell fusion assay with KSHV gH-ASAELAAN/gL/RRV gB expressing effector cells. The sera of Group 1, 2, 3 and 4 inhibited significantly compared to the control sera (*p* < 0.05; one-way ANOVA). Error bars represent the standard deviation. The experiments (**D**–**F**) were performed three times.

Our results with regard to neutralization and receptor binding so far suggested inhibition of receptor binding as a neutralization mechanism. In other herpesviruses, gH/gL controls the fusion activity of gB and does not necessarily interact with cellular receptors. The binding to cellular receptors, which provides a fusion trigger, is in those cases mediated by specialized viral GPs, such as gD—the prototypical receptor binding protein in herpes simplex viruses—that activates gH/gL to then activate gB [16]. Compatible with such receptor-independent functions of gH/gL, we had previously observed that KSHV could also enter cells in an Eph-independent manner and that loss of the interaction with receptors from the Eph family can be overcome by increasing the amount of virus in the inoculum [27]. To deconvolute inhibition of receptor binding from other mechanisms, we made use of a novel KSHV mutant (KSHV gH-ASAELAAN) that is similar to an already described gH mutant, KSHV gH-ELAAN [27] and loses interaction with Eph receptors. In addition, gH-ASAELAAN at best minimally incorporates gL into the gH/gL complex (Figure 4A). 

We used this mutant to discriminate between the effects of antibody responses directed to the receptor-binding domain I of gH/gL, that is formed by gH and gL through folding together, and the effects of antibody responses directed to the rest of the gH ectodomain. The gH-ASAELAAN mutant was generated by combining gH mutations that we had previously found to individually decrease either EphA2 binding (E52A, F53A) or gH/gL complexation (L47A, I49A) [27], which together caused drastically reduced or abrogated gL incorporation into the gH/gL complex (Figure 4A) in addition to loss of binding to EphA2 as evidenced by the inability of soluble EphA2 or ephrin ligands to block infection of KSHV bearing the gH-ASAELAAN mutations (Figure 4B). The loss of gL binding through mutations in gH residues L47 and I49 is compatible with their location at the binding interface with gL [37]. The ASAELAAN mutation also led to increased accumulation of gL in cell lysates and shifted glycosylation of gH, a phenomenon also observed for, e.g., pseudorabies virus gH in the absence of gL [38]. The structure of domain I of gH/gL is conceivably much more disturbed in the gH-ASAELAAN mutant than in the previously described gH-ELAAN mutant. gL forms a large interaction surface with, e.g., EphA2 [37] and possibly other Ephs. While Eph dimerization and receptor usage was abrogated through mutation of E52 [37] or E52 and F53 [20,33,39], we chose the gH-ASAELAAN mutant to exclude any remaining contacts of gL to Ephs or other potential receptors and to analyze the contribution of gH alone, similar to a ∆gL mutant that we have analyzed for the related rhesus monkey rhadinovirus (RRV) [29,40]. A true KSHV ∆gL mutant is not trivial to construct because of several overlapping, spliced transcripts that span from orf47, which encodes gL, to orf45, and that are critical for KSHV lytic replication [41]. 

The KSHV gH-ASAELAAN mutant was equally detargeted from Eph receptors as the previously described gH-ELAAN mutant (Figure 4B), and was equally resistant to both soluble EphA2-Fc decoy receptor and to ephrinA4-Fc as a competitor for EphA receptors. As described previously, Eph-detargeted KSHV mutants are approximately 4–9 fold less infectious, depending on the target cell, which can be overcome by increasing the amount of input virus [27]. 

Interestingly, sera from all immunization regimens neutralized the KSHV gH-ASAELAAN virus at similar levels (Figure 4C), including the sera from Group 6 that was immunized with the gHecto-ferritin/gL construct and that exhibited low EphA2 blocking activity (Figure 3D), compatible with the finding that inhibition of Eph binding does not play a role for KSHV gH-ASAELAAN. Maximum inhibition averaged across all groups was approximately 84% for the KSHV gH-ASAELAAN virus compared to approximately 91% for the wildtype virus.

### 3.5. Sera Raised against gH/gL Inhibit Activation of gB for Fusion

As inhibition of binding to Eph family receptors was obviously not the only mechanism of neutralization, we tested the different anti-gHecto/gL sera in a fusion assay together with the heterologous gB from the related RRV. Use of this heterologous gB is necessary as KSHV gB is not robustly activated for cell–cell fusion by KSHV gH/gL [21,39], but RRV gB is [28]. RRV gB alone was not fusogenic and barely so in combination with KSHV gH as opposed to strong fusion activity when paired with KSHV gH/gL (Figure 4D). In direct comparison to WT KSHV gH/gL, gH-ASAELAAN, which does not readily complex with gL, together with KSHV gL and with RRV gB, exhibited drastically reduced fusion activity, in keeping with results reported for gH-ELAAN [39] and with what would be expected without gL in the complex. We clearly observed inhibition of cell–cell fusion by sera from all groups (Figure 4E,F). Fusion triggered by the gH-ASAELAAN construct was also inhibited (Figure 4F), even if effect sizes were much smaller, at least in part because of the initially much lower level of activity of the mutant gH/gL complex. Nevertheless, these results demonstrate that KSHV gH/gL enables gB to fuse also in an Eph-independent manner and that antibodies against gH/gL interfere with this activation.

## 4. Discussion

The data presented here refute our hypothesis that removal of glycosylations from gH/gL would elicit more potently neutralizing antibodies by exposing otherwise potentially shielded epitopes during the prime immunization. The gL-gHecto fusion protein, on the other hand, induced a strong neutralizing response, and because it can be expressed from a single cDNA, this construct is a good candidate for nucleic acid-based immunization strategies, like a vector or mRNA lipid nanoparticles. Our results using the KSHV gL-gHecto antigen are consistent with data reported for the equivalent EBV construct, which was shown to be a potent immunogen [35]. Finally, we employed a ferritin-based approach that is based on the ability of ferritin to self-assemble into nanoparticles that display multiple copies of an antigen on the surface. Such particles carrying EBV gH/gL were reported to elicit potent immune responses [36]. We did not observe a strong response, neither with regard to reactivity against gH/gL, as measured by ELISA, nor with regard to neutralization of WT virus. Surprisingly, this qualitative difference compared to sera from the other groups was limited to the capability to inhibit EphA2 receptor binding (Figure 3C) and neutralization of KSHV with a WT gH/gL complex (Figure 3E), but did not extend to neutralization of the KSHV gH-ASAELAAN mutant. The observation that sera from all groups neutralized a KSHV carrying a mutant gH (gH-ASAELAAN) that does not bind EphA2 (Figure 4C) has twofold implications. First, the ferritin construct, which was less potent at eliciting antibodies blocking interactions with EphA2, likely through targeting gH domain I, was as potent as the WT gH/gL complex at eliciting neutralizing antibodies against KSHV gH-ASAELAAN, likely through eliciting antibodies targeting the gH domains II-IV, which do not participate in Eph-binding [37]. Whether comparatively weaker inhibition of the gH/gL interaction with EphA2 by sera from gHecto-ferritin/gL-immunized mice might be due to a less stable complexation with gL in this construct or represents a possible steric effect the nanoparticle may have on the gH domain I and gL, remains unclear, but it suggests that immunization strategies that work for the related EBV may not work optimally for KSHV. At least gL incorporation was robust (Figure 2B) and can likely be excluded as the reason for the different immune responses. Second, our results demonstrate that gH fulfills functions that are independent of Eph receptor binding. While not entirely surprising, the KSHV gH/gL complex functions in infection and also in the fusion both in an Eph receptor-dependent and in a receptor-independent manner, under the assumption that results with the heterologous RRV gB are representative for KSHV gB. According to our results with the gH-ASAELAAN mutant and our recent findings with a gL deletion mutant of the related RRV [29,40], it is likely that gH can perform some of these functions, in particular in membrane fusion, without gL. This highlights the redundancy of the KSHV entry process and strongly suggests that targeting multiple sites on gH/gL and possibly also on other proteins with a potential vaccine may be ideal for protection.

## Figures and Tables

**Table 1:** Oligonucleotides.

Oligonucleotides	Sequence
gL N118Q for	AAGCCACCACAGCCGACAG
gL N118Q rev	GAAAGCCCACTGTATAGGCGGTC
gL N141Q for	AGCGGACCGGCTCTGTGAG
gL N141Q rev	GCATGGCCTTGCCCACAGAG
KSHVgH_N46Q_for	GCTGAGCATCGAGCTGGAATTC
KSHVgH_N46Q_rev	TGAGTCCGGCCGTTAATCAGC
KSHVgH_N54Q_for	GGGGACCTCCTTCTTTCTGAATTG
KSHVgH_N54Q_rev	TGGAATTCCAGCTCGATGCTCAG
for (gH-22)	AGCCCCGCAAGTCAGTGAG
rev (gH 23-)	ACTGGGGCTCTGCCTACC
for ([gH-22] gL 21-)	CTCACTGACTTGCGGGGCTTATGTCGCTCTGCCCTGTTGTG
rev (gL-167 [gH 23-])	GTGGTAGGCAGAGCCCCAGTTTTCCCTTTCTGCCCTGCGTG
ferritin_for1KSHV	ACAGAAGGCGCGCCGCTTCTGAGAGTCAAGTCCGGCAAC
ferritin_rev	TTACCTTCGAAGGGCCCTTATCAGGACTTACGTGATTTCGC
KSHV_gH_for	AGAAGCGGCGCGCCTTCTG
gH_rev	TAAGGGCCCTTCGAAGGTAAGCC
Ax25-ASAELAANs	GCATCCGCTGAACTGGCAGCAAAC
Ax25-ASAELAANas	GTTGGTTCTCCCATTGATGAGCTGCG
EphA2-436	CTGGTTGATGCTGACACTGGC
EphA2 rev2	CATCATCACCATCACCATGAGTAAACC

**Table 2:** Antibodies.

Target	Details
V5-tag	Mouse, Bio-Rad, 1:1000, (secondary: Dianova, donkey anti-mouse 1:10,000)
DYKDDDDK (FLAG) tag	rabbit, Cell Signal Technology (D6W5B), 1:1000, (secondary: Dianova, goat anti-rabbit 1:10,000)

## Data Availability

Not applicable.
